# The Role of Machine Learning in Cognitive Impairment in Parkinson Disease: Systematic Review and Meta-Analysis

**DOI:** 10.2196/59649

**Published:** 2025-03-14

**Authors:** Yanyun Wu, Yangfan Cheng, Yi Xiao, Huifang Shang, Ruwei Ou

**Affiliations:** 1 Department of Neurology West China Hospital of Sichuan University Chengdu China

**Keywords:** Parkinson disease, cognitive impairment, machine learning, systematic review, meta-analysis

## Abstract

**Background:**

Parkinson disease (PD) is a common neurodegenerative disease characterized by both motor and nonmotor symptoms. Cognitive impairment often occurs early in the disease and can persist throughout its progression, severely impacting patients’ quality of life. The utilization of machine learning (ML) has recently shown promise in identifying cognitive impairment in patients with PD.

**Objective:**

This study aims to summarize different ML models applied to cognitive impairment in patients with PD and to identify determinants for improving diagnosis and predictive power for early detection of cognitive impairment.

**Methods:**

PubMed, Cochrane, Embase, and Web of Science were searched for relevant articles on March 2, 2024. The risk of bias was assessed using the Quality Assessment of Diagnostic Accuracy Studies-2 (QUADAS-2). Bivariate meta-analysis was used to estimate pooled sensitivity and specificity results, presented as odds ratio (OR) and 95% CI. A summary receiver operator characteristic (SROC) curve was used.

**Results:**

A total of 38 articles met the criteria, involving 8564 patients with PD and 1134 healthy controls. Overall, 120 models reported sensitivity and specificity, with mean values of 71.07% (SD 13.72%) and 77.01% (SD 14.31%), respectively. Predictors commonly used in ML models included clinical features, neuroimaging features, and other variables. No significant heterogeneity was observed in the bivariate meta-analysis, which included 12 studies. Using sensitivity as the metric, the combined sensitivity and specificity were 0.76 (95% CI 0.67-0.83) and 0.83 (95% CI 0.76-0.88), respectively. When specificity was used, the combined values were 0.77 (95% CI 0.65-0.86) and 0.76 (95% CI 0.63-0.85), respectively. The area under the curves of the SROC were 0.87 (95% CI 0.83-0.89) and 0.83 (95% CI 0.80-0.86) respectively.

**Conclusions:**

Our findings provide a comprehensive summary of various ML models and demonstrate the effectiveness of ML as a tool for diagnosing and predicting cognitive impairment in patients with PD.

**Trial Registration:**

PROSPERO CRD42023480196; https://www.crd.york.ac.uk/PROSPERO/view/CRD42023480196

## Introduction

Parkinson disease (PD) is the second most common progressive neurodegenerative disease. It has gained much attention from scientists due to its rising rates of disability and death [1]. The cause of PD involves the clumping of misfolded α-synuclein in Lewy bodies in neurons. It also involves mitochondrial problems, neuroinflammation, and oxidative stress. These processes lead to irreversible cellular damage and neuronal loss. Several factors influence the development of PD, including environmental factors such as exposure to pesticides, lifestyle factors, such as tobacco consumption and sedentary behavior, genetic mutations, such as *GBA*, *LRRK2*, *PARK*, and *SNCA*, as well as demographic factors like advancing age and male gender [2]. Yet, the cause of PD remains unknown.

The cardinal motor symptoms of PD include bradykinesia, rigidity, rest tremor, and postural instability. In addition, nonmotor symptoms such as cognitive impairment, sleep disorders, and autonomic dysfunction are commonly observed. A recent study has stressed the rising importance of nonmotor symptoms in the later stages of the disease. It has highlighted their key role in PD management [3].

Cognitive impairment in PD is marked by problems in many cognitive domains, including attention, memory, executive function, language, and visuospatial function [4]. The spectrum of cognitive impairment in PD ranges from mild cognitive impairment (MCI) to PD dementia (PD-D) [5]. The definition and progression of PD-MCI and PD-D exhibit significant heterogeneity. PD-MCI is defined as a decline in cognition that deviates from what is expected based on the patient’s age and education level but does not meet the criteria for impaired cognitive function [6]. MCI can occur in the early stages of the disease, even before the onset of motor symptoms, and may be overlooked. In contrast, dementia typically appears in the advanced stages of PD and causes impairments across multiple cognitive domains. These deficits are severe enough to disrupt the daily life of motor symptoms. Patients who meet specific criteria [7] can be diagnosed with probable or possible PD-D. Researchers have identified MCI as a significant risk factor for the development of dementia in individuals with PD [8]. A meta-analysis has shown differences in biomarkers and brain imaging between patients with PD-D and those with PD-MCI [9].

Recently, the use of artificial intelligence has grown, specifically machine learning (ML). ML is a subfield of artificial intelligence. It focuses on developing algorithms and statistical models that allow computers to perform tasks without explicit programming. The main goal of ML is to enable computers to learn from data and make predictions or decisions based on that learning [10,11]. ML has 4 types: supervised, unsupervised, semisupervised, and reinforcement learning. ML can be broadly classified into 2 main categories: supervised and unsupervised learning. The former encompassed a range of models, including support vector machine (SVM), random forest, K-nearest neighbors, and naive Bayes, among others. In contrast, unsupervised learning involves clustering, dimensionality reduction, and so on. They differ in their use of labeled or unlabeled data to train models. Semisupervised learning is a hybrid approach. It combines supervised and unsupervised learning. Reinforcement learning does not require data with labels; instead, it learns from experiences by interacting with the environment, observing, and responding to results.

Current evidence has proven that ML is useful in predictive analytics for medicine. It can analyze complex datasets, including clinical data, genetic information, and imaging features to aid in the early detection and diagnosis of diseases. Besides, it can assess the risk of progression and prognosis. For example, in conditions like Alzheimer disease and PD, ML models can find patterns in imaging data for more accurate diagnosis. The latest research trained an XGBoost (extreme gradient boosting) model to predict PD-associated genes using genomic, transcriptomic, and epigenomic data from brain tissues and dopaminergic neurons [12]. ML has been used as a valuable technique to predict the high risk of disease conversion [13]. Vast health data is available, including clinical, genetic, imaging, and biomarker data. They can be used to diagnose and predict prognosis, rather than being based on symptoms and performed by specialist neurologists.

In this review, our goal is not to give a full literature review of articles applying ML to clinical problems, nor do we aim to delve into the complex mathematical details of numerous ML methods. Instead, we focus on summarizing the existing ML models. They are used to diagnose PD with cognitive impairment and to predict cognitive decline as the disease progresses. We also aim to list predictors that may help in diagnosis, progression, and prognosis in patients with PD with cognitive impairment.

## Methods

### Search Strategy

Two authors (WYY and XY) independently searched PubMed, Cochrane, Embase, and Web of Science databases for relevant articles using the keywords “Parkinson’s disease,” “Machine Learning,” and “cognitive decline” in different combinations (Table S1 in Multimedia Appendix 1). The included studies were from the start of the database until March 2, 2024. The review was reported according to the PRISMA (Preferred Reporting Items for Systematic Review and Meta-Analyses) statement. It is registered in PROSPERO (International Prospective Register of Systematic Reviews; number CRD42023480196).

### Eligibility Criteria and Study Selection

Studies were eligible if they aimed to diagnose or predict the cognitive impairment in PD. The target condition was the normal cognition (PD-NC), PD-MCI, and PD-D. ML models were carried out to predict the target condition. Studies were eligible if these reported data on the following: true positive (TP), true negative (TN), false positive (FP), false negative (FN), sensitivity, specificity, accuracy, positive predictive value, or negative predictive value, and others. In case of not reporting TP, TN, FP, or FN, these were calculated from known variables (sensitivity and specificity).

The articles retrieved from the electronic databases were imported into EndNote (Clarivate) for further analysis. Duplicates, reviews, dissertations, cases, and conference abstracts were systematically eliminated through an automated process. Two authors (WYY and XY) independently screened the titles and abstracts of the rest of the records. Any disagreements were decided by the third author (CYF). Following this step, the full text of the remaining studies was downloaded and carefully reviewed, resulting in the final selection of relevant articles.

The data collected from the chosen studies included publication details such as title, first author, and year of publication, as well as study information such as study design, whether it was conducted at single or multiple centers, diagnostic criteria for PD and cognitive impairment, ML models employed, predictors used, and value index assessed.

### Risk of Bias

There is no widely accepted checklist for assessing the quality of diagnostic ML papers. We chose combined criteria that had been used in a previous report [14]. Two review authors (WYY and XY) independently assessed the risk of bias based on the Quality Assessment of Diagnostic Accuracy Studies-2 (QUADAS-2) [15], and any disagreements were resolved through the third author (CYF). If one of the questions was scored at a high risk of bias, the domain was scored at a high risk of bias. At least one domain at high risk of bias resulted in an overall score of high risk of bias, and only one domain scored as unclear risk of bias resulting in an overall score of unclear risk of bias for that paper.

### Performance Metric and Meta-Analysis

Due to the diversity of models and predictors under study, models were categorized by type (random forest, neural network, etc) irrespective of variable selection procedures in the study. Similarly, predictor variables were grouped for analysis. For example, demographic features (such as age, gender, family history, and education), clinical features (such as motor symptoms, nonmotor symptoms, and various scale tests, such as Mini-Mental Status Examination [MMSE] and Montreal Cognitive Assessment [MoCA]), neuroimaging features (such as magnetic resonance imaging [MRI] and single photon emission computed tomography features), biofluid features (such as -synuclein [-syn], amyloid β [Aβ], and phospho-microtubule-associated protein tau [p-tau] in cerebrospinal fluid and blood plasma), genetic features (such as *GBA*, *LRRK2*, and single-nucleotide polymorphism [SNP] variants), and quantitative electroencephalography.

Given that classification algorithms in ML can indeed be considered as a diagnostic test, and that the results included in the article, ultimately, we used bivariate meta-analysis [16] to estimate the pooled sensitivity and specificity results, along with their corresponding 95% CIs. Estimated sensitivity and specificity were represented as forest plots. A summary receiver operator characteristic (SROC) curve was generated to evaluate the accuracy of ML for the prediction of cognitive impairment in patients with PD [17]. The test of heterogeneity was assessed using the Cochrane Q-test and *I*^2^ test [18]. Heterogeneity was defined as significant if the Q-test results showed *P*<.05 or *I*^2^>50%. Potential publication bias was estimated using Deeks’ funnel plot. In the event of bias, it may be evidenced by the visual asymmetry observed in conventional funnel plots. A Fagan nomogram was used to identify the maximum pretest and posttest likelihood. We also conducted sensitivity analysis by removing studies with a high risk of bias to identify potential sources of heterogeneity. Considering not all studies reported the results of both the train and validation sets, we also analyzed the single set. Statistical analyses were performed using GraphPad Prism 9.5 (GraphPad Software, Inc) and Stata (version 17; StataCorp). The level of significance was set at *P*<.05.

## Results

### Study Selection

**Figure 1:**
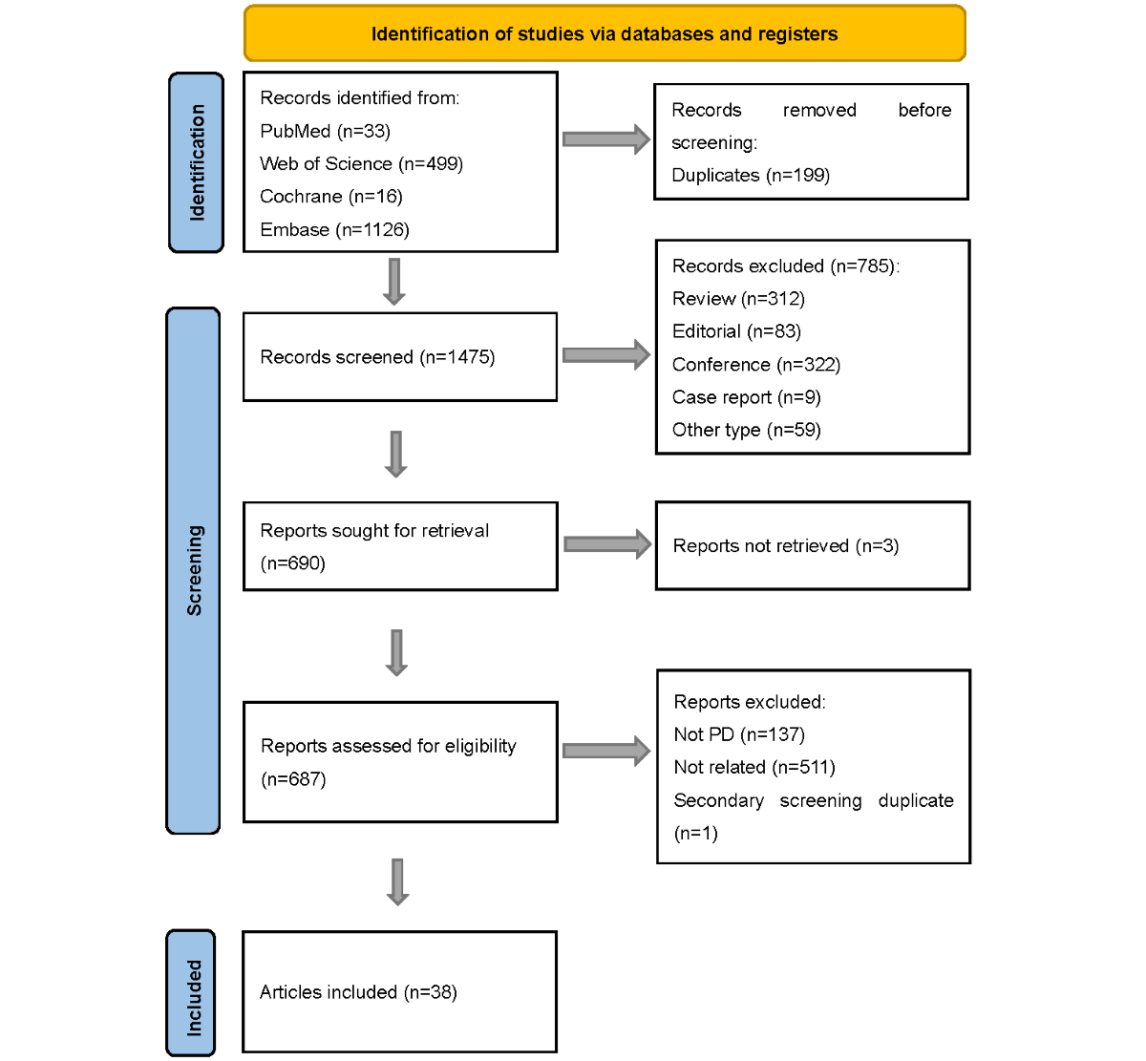
Flowchart illustrating the process of searching for screening and inclusion. PD: Parkinson disease.

Figure 1 shows the literature selection and filtering process following the PRISMA 2020 guidelines [19]. A total of 1674 records were identified from 4 databases. After removing 199 duplicates, 1475 records were screened, with 785 excluded for being unrelated (reviews, editorials, conference abstracts, case reports, etc). Of the 690 records sought for retrieval, 3 were not obtained. Finally, 687 reports were assessed for eligibility, with 137 excluded for not being related to PD and 511 excluded for being irrelevant. Finally, 38 studies [20-57] published between 2013 and 2024 were included in the qualitative analysis, from which 12 studies [20-31] were included in the meta-analysis. A total of 9698 participants were involved in the current study, comprising 8564 patients with PD and 1134 healthy controls in qualitative analysis. Additional information can be found in Table S2 in Multimedia Appendix 1 [20-57].

### Risk of Bias

Table S3 in Multimedia Appendix 1 shows the risk of bias status of each included article. A total of 18 [24,27,29,32,33,39,42-47,51,52,54-57] out of 38 (47%) studies [20-57] were scored as high risk of bias, 9 [30,31,37,38,40,41,49,50,53] out of 38 (23%) studies were scored as unclear risk of bias, and the rest of the studies were low risk of bias. Studies scored a high risk of bias for failing to describe their study population (patient selection), losing the gold standard for diagnosing cognitive impairment (reference standard).

### ML Models and Predictors

In the 38 studies [20-57], 172 models were constructed with different ML models and predictors. However, 142 models were subjected to analysis, with accuracy being the primary outcome. These included 45 SVM models, 29 random forest models, 9 K-nearest neighbors models, 18 naive Bayes models, and other supervised learnings. Also, we included 3 semisupervised models that used SVM with principal component analysis PCA and 2 neural networks. The overall mean accuracy of 142 models was 74.32% (SD 10.25%). Of the 89 models that reported area under the receiver operating characteristic curve, the mean accuracy was 0.74 (SD 0.12). Also, 120 models reported sensitivity and specificity, with mean values of 71.07% (SD 13.72%) and 77.01% (SD 14.31%), respectively.

**Figure 2:**
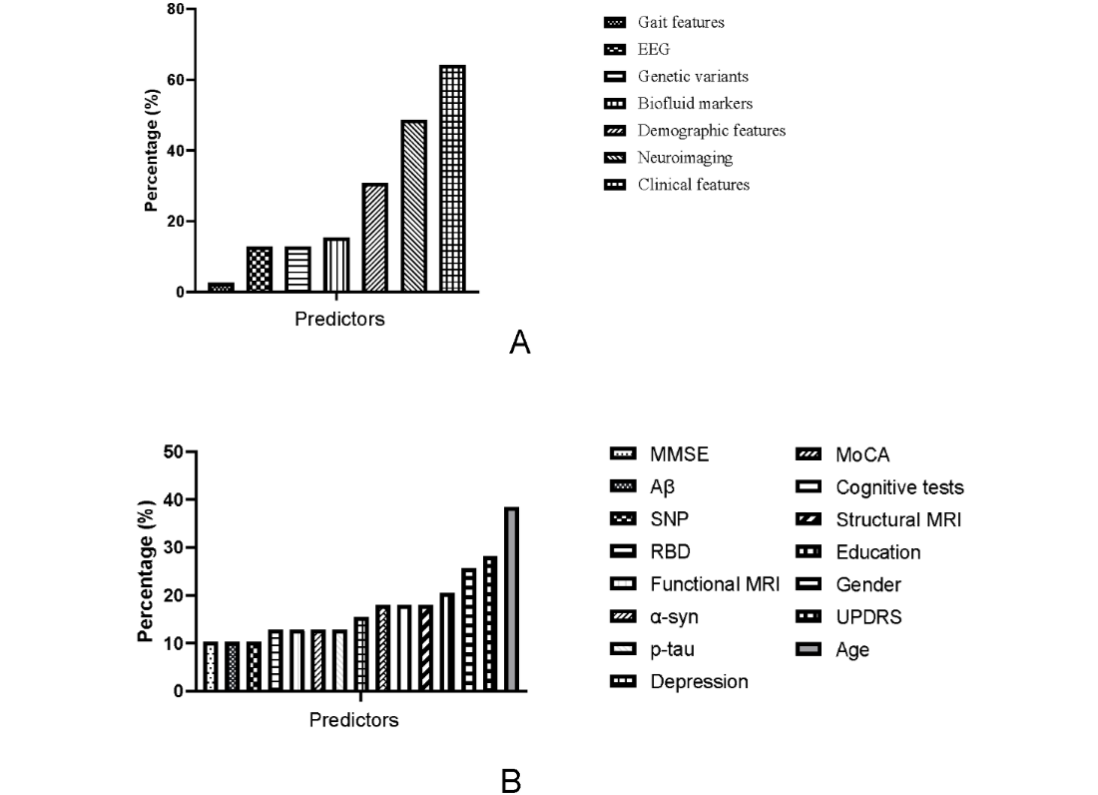
Predictors used for diagnosing and predicting cognitive impairment in Parkinson disease in included models. EEG: electroencephalography; MMSE: Mini-Mental Status Examination; MoCA: Montreal Cognitive Assessment; MRI: magnetic resonance imaging; p-tau: phospho-microtubule-associated protein tau; RBD: rapid eye movement sleep behavior disorder; SNP: single nucleotide polymorphism; UPDRS: Unified Parkinson’s Disease Rating Scale; α-syn: α-synuclein.

This study discovered the predictors used to diagnose and predict cognitive impairment in PD (Figure 2A). Clinical features have the highest percentage, followed by neuroimaging and demographic features. Other predictors such as biofluid markers, genetic variants, electroencephalography, and gait features show a more balanced or lower percentage representation. The top-used predictors were summarized in 142 models (Figure 2B). The demographic and clinical characteristics that emerged as the most significant predictors included age, gender, and education level, as well as the Unified Parkinson’s Disease Rating Scale (UPDRS), rapid eye movement sleep behavior disorder, MMSE, and MoCA. Furthermore, additional neuroimaging and biomarkers have been identified as potentially contributing to the prediction process. Overall, 116 out of 142 models used a single type of feature to diagnose, with a mean accuracy of 73.42% (SD 10.24%). Nearly 18% of models used multiple types of features combined, with a higher accuracy of 78.57% (SD 9.2%). In the single type of model, the predictors with the highest accuracy were neuroimaging features, achieving an accuracy of 78.78% (SD 11.48%). Furthermore, 13 articles used longitudinal data to predict cognitive impairment or conversion, with the majority of predictors being neuroimaging features and biofluid markers.

### Meta-Analysis

**Figure 3:**
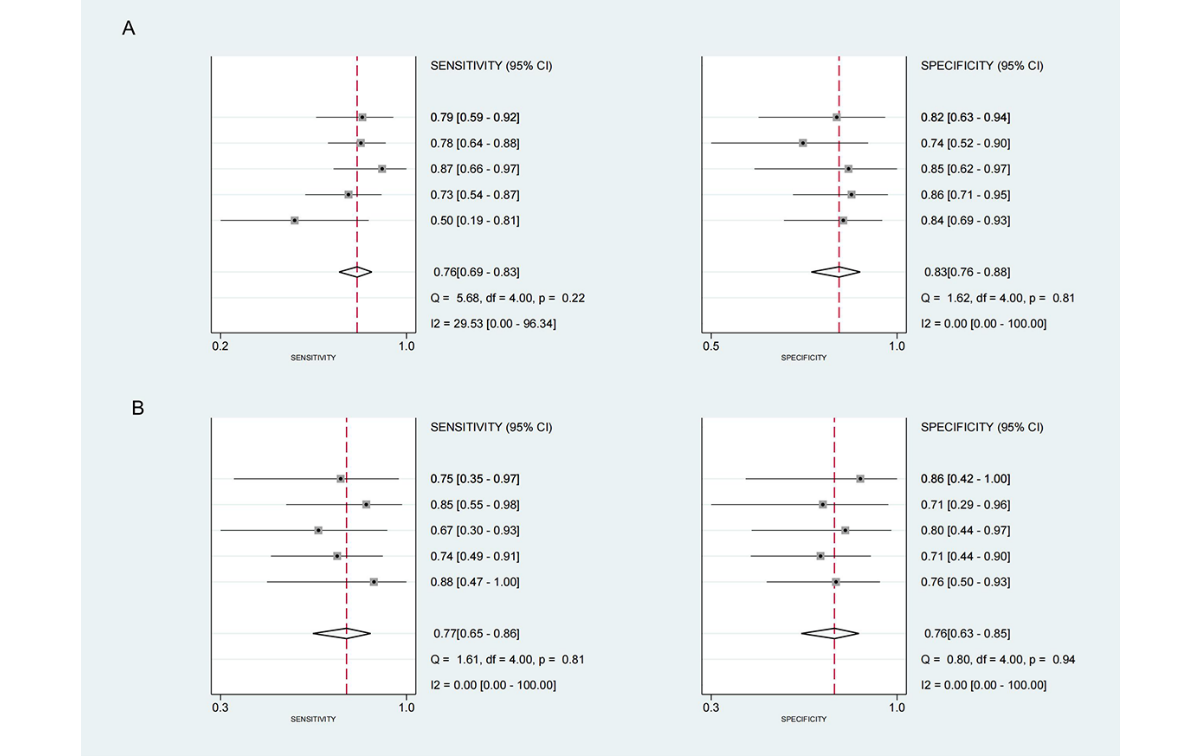
Forest plots for sensitivity and specificity in studies with both train and validation sets.

**Figure 4:**
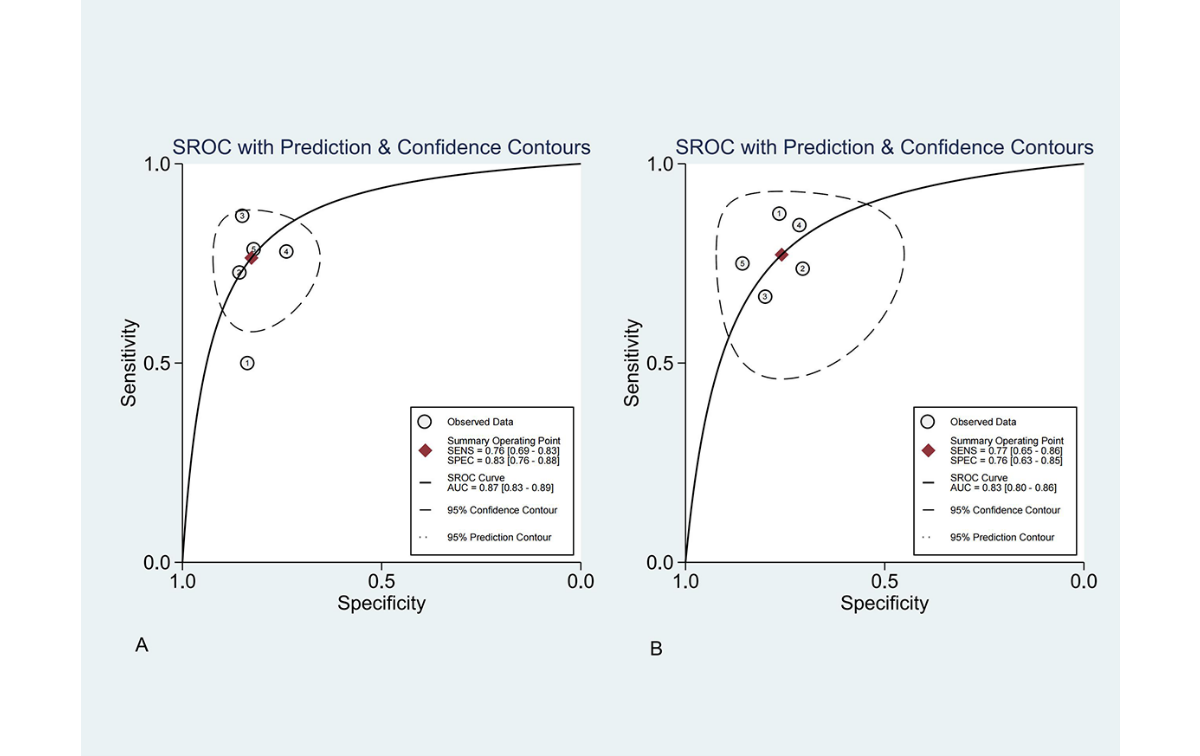
Summary receiver operating characteristic curve for sensitivity and specificity in studies with both train and validation sets. AUC: area under the curve; SROC: summary receiver operating characteristic.

Due to limited reporting of TP, TN, FP, FN, sensitivity, and specificity, only 12 studies were conducted for meta-analysis [20-31]. For studies with multiple models, we prioritized the one with the highest accuracy, reported in 63% (24/38) of the articles, to represent optimal model performance. A total of 7 studies [25-31] reported outcomes in the train set or validation set, while 5 studies [20-24] reported outcomes in both the train set and validation set. Forest plots for sensitivity and specificity are shown in Figure 3 for the train and validation set, respectively, and Figure S1 in Multimedia Appendix 1 for the single set (train or validation set). The training set sensitivity exhibited different results in the Q-test (Q=5.68, *P*=.22) and *I*² test (*I*²=29.53). Other sensitivity and specificity values showed no heterogeneity. Using sensitivity, the combined sensitivity and specificity were 0.76 (95% CI 0.67-0.83) and 0.83 (95% CI 0.76-0.88), respectively. When using specificity, the combined values were 0.77 (95% CI 0.65-0.86) and 0.76 (95% CI 0.63-0.85), respectively. The area under the curves of the SROC were 0.87 (95% CI 0.83-0.89) and 0.83 (95% CI 0.8-0.86), respectively (Figure 4; Figure S2 in Multimedia Appendix 1). Fagan plots were constructed to illustrate the pretest and posttest probability of ML predicting cognitive impairment in patients with PD (Figures S3 and S4 in Multimedia Appendix 1). The Deeks’ test showed no significant publication bias for ML (Figures S5 and S6 in Multimedia Appendix 1).

### Sensitivity Analysis

According to the risk of bias analysis, 3 articles scored high. After excluding these studies, all results exhibited no statistically significant heterogeneity, for the sensitivity of the training set. The results indicated a moderate degree of heterogeneity between studies (*I*²=45.45). However, the results suggested no statistically significant heterogeneity between studies in the Q-test (Q=5.5, *P*=.14; Figure S7 in Multimedia Appendix 1). The sensitivity, specificity, and SROC were consistent with the original analysis (Figure S9 in Multimedia Appendix 1). In the single set, there exists high heterogeneity both in sensitivity (Q=14.6, *P*=.01; *I*²=72.59) and in specificity (Q=96.07, *P*<.01; *I*²=95.84; Figure S8 in Multimedia Appendix 1). The results were consistent with the original analysis, with high sensitivity and specificity, and good overall diagnostic accuracy (Figures S9 and S10 in Multimedia Appendix 1). Fagan plots are shown in Figures S11 and S12 in Multimedia Appendix 1. The Deeks’ test also found no significant publication bias for ML (Figures S13 and S14 in Multimedia Appendix 1).

## Discussion

### Principal Findings

This meta-analysis provided new insights into the efficacy of ML in diagnosing cognitive impairment in patients with PD. The average accuracy of 142 models was 74.32%, with 120 models reporting mean sensitivity and specificity values of 71.07% (SD 13.72%) and 77.01% (SD 14.31%), respectively. No significant heterogeneity was found among the sensitivities and specificities reported by the analyzed included in our meta-analysis. Nearly 50% of the included studies had a high risk of bias affecting applicability. A total of 12 studies [22,31,36,37-44,53] explored the cognition changes in a longitudinal cohort of patients with PD. Although complete data were not obtained in this study, we could conclude that the ML can predict potential cognitive impairment at baseline and forecast changes in cognitive state.

According to the QUADAS-2, most of the studies had high or unclear risk in the patient selection and reference standard. Part of the studies lacked detailed patient source descriptions. However, these articles were not excluded due to the use of Movement Disorder Society diagnostic criteria for cognitive impairment in patients with PD [7,58]. Many studies faced similar issues in patient selection, and complete exclusion would significantly reduce the available data. The diverse metrics used to assess ML resulted in incomplete reporting of expected metrics, increasing bias risk. These problems may lead to unstable meta-analysis results and make it difficult to provide meaningful conclusions. Furthermore, the low heterogeneity between studies limits the values of sensitivity analyses.

The meta-analysis of 12 studies [20-31] demonstrated that ML models exhibited greater consistency in terms of sensitivity and specificity in the training and validation sets. In addition, no heterogeneity or publication bias was observed, suggesting that the ML model exhibits superior performance in predicting cognitive impairment in patients with PD. However, further studies are essential to validate its clinical applicability.

Studies with a high risk of bias were excluded, and the results were found to be consistent with those of the original analyses. However, the *I*² in the sensitivity of the train set exhibited a slight increase, while the Q-test suggested no statistically significant heterogeneity. The sensitivity analysis demonstrated the credibility and robustness of the results. The results of the remaining 9 studies [20-23,25,26,28,30,31] remained stable compared to the original analysis and showed high sensitivity and specificity of the ML to diagnose and predict cognitive impairment in patients with PD. While, due to the limited number of studies included in the meta-analysis, further exploration was required.

### ML Models and Predictors

A recent review divided ML into traditional ML (including supervised and unsupervised ML), and ML methods based on neural networks (such as convolutional neural networks and recurrent neural networks) [59]. The majority of research still focuses on traditional ML in this review, possibly because supervised learning still has advantages in ML algorithms and processed data. First, supervised learning can be further divided into classification and regression based on their tasks. It can differentiate between patients with cognitive impairment and those with mild cognitive normality and also can identify patients with MCI in PD from those who can be diagnosed with dementia. Unsupervised learning is primarily used for clustering, aiming to discover inherent groupings in the data. It is limited to categorizing patients into distinct groups by reducing the dimensionality of their shared characteristics. It cannot offer a definitive diagnosis due to the absence of labeled features. Second, although unsupervised learning is proving valuable in reducing the cost of labeling data and automatically identifying data structures and patterns, the results still require manual interpretation and analysis for interpretability [60].

Determining the superiority of ML methods is challenging. With the development of imaging technology and the increasing costs of data collection, unsupervised learning, deep learning, and neural networks may become more prevalent in the future. Notably, semisupervised learning also showed great potential [61]. Chen et al [32] combined SVM with principal component analysis to successfully extract 6 features from 32 predictors, improving the performance over the simple SVM model. Other models use unsupervised learning to cluster this unlabeled data and then use supervised learning for longitudinal prediction [33]. Further research and discussion are needed to assess the accuracy and effectiveness of these different models.

We found that cognitive tests such as the MMSE and the MoCA were mostly used as predictors. Meanwhile, the UPDRS score as a specific feature that can reflect the patient’s severity of motor and nonmotor symptoms was highly recommended as a potential predictor. In addition, with the advancement of neuroimaging techniques, not only traditional neuroimaging, such as cortical thickness but also functional MRI was widely used to diagnose and predict disease progression, including Fluorine-18 fluorodeoxyglucose positron emission tomography, which can reflect the metabolism in the brain and diffusion tensor imaging which can show the damages to brain connectivity. On the other hand, demographic features such as older age, male sex, and lower education, which were considered as highly risk factors to cause PD, were highly ranked. Biofluid markers, total-tau, p-tau, Aβ, and -syn detected in the cerebrospinal fluid were the most popular approaches and were deemed as the vital factors to show the prognosis. Furthermore, the rise of genomics has become a crucial factor in diagnosis and prediction. The growing popularity of genome-wide association studies research has made SNP variation a focal point of interest, more and more SNP variants that count were found to cause PD. The *APOE* gene, which is the risk factor in AD, is also a potential predictor to predict cognitive impairment in PD. This review also shows that the performances of ML vary depending on its feature input. Some studies have found inconsistent accuracy when using single or multiple types of features. Some studies have reported greater accuracy in models with a single type of feature model [34], while others have concluded that the models incorporating multiple types of features are more effective [21,24,26]. Despite the remaining discrepancies in the existing research, integrating multiple types of features for predictive models appears to yield better results overall.

This review also summarized the longitudinal studies on predicting cognitive impairment and conversion in PD [22,31,36-39]. We discovered that neuroimaging features and biofluid markers are robust predictors. With the advancement of imaging technology, such as functional MRI and magnetic resonance spectroscopy, and their noninvasive detection capabilities, neuroimaging has become mainstream today. Neural networks and deep learning are especially focused on applications in imaging. Numerous research studies have suggested that biomarkers such as Aβ, -syn, and tau, play an essential role in the pathologic changes in PD. The detection of these biomarkers in fluids such as cerebrospinal fluid and blood becomes a potential approach to improving the accuracy of ML in diagnosing and predicting the status of cognitive impairment and disease progression.

A personal review proposed new diagnostic criteria: a 3-component system (SynNeurGe). It includes the harmful α-synuclein (S) accumulated in tissues or cerebrospinal fluid, evidence of neurodegeneration (N) in imaging, and the disease-causing gene variants (G) for PD. They are associated with clinical symptoms, defined either by a single highly specific clinical feature or by multiple less specific clinical features. A biological classification will aid in both basic and clinical research and bring the field closer to the precision medicine needed to develop disease-modifying therapies [62]. Consequently, a universally applicable ML model that integrates these significant variations can lead to personalized diagnosis and prediction, providing immense value for early detection, intervention, and management.

### Strengths and Limitations

A large number of studies have investigated the diagnostic accuracy of ML to diagnose cognitive impairment in patients with PD. With the increasing number of deep learning and detection approaches, a more comprehensive exploration of ML was conducted. We used bivariate analysis to show more robust results. However, there are still several limitations in this review. First, we excluded nonpublished data in the meta-analysis. Due to heterogeneity in intervention methods and outcome measures between studies, it was not possible to synthesize all articles included using meta-analysis. Second, several studies used Parkinson’s Disease Progression Markers Initiative datasets, as they focus on the longitudinal cognitive change. However, we did not analyze the predictive power of ML in the follow-up cohort [22,31,36,38-46]. We are also limited to analyzing the optimum models and the predictors. Further investigation is required to determine the most effective model or predictors for identifying patients with PD who have cognitive impairment, predicting the disease progression (including conversion to dementia), and providing personalized treatment options.

### Conclusions

ML algorithms have been proven to be highly effective in diagnosing cognitive impairment in patients with PD, especially MCI, compared to normal cognitive function. Furthermore, ML holds significant potential for predicting the cognitive decline in patients with risk factors and the transition from MCI to dementia.

## Appendix

Multimedia Appendix 1 Additional figures and tables.

Multimedia Appendix 2 PRISMA checklist.

## Abbreviations

-syn: -synuclein
Aβ: amyloid β
FN: false negative
FP: false positive
MCI: mild cognitive impairment
ML: machine learning
MMSE: Mini-Mental Status Examination
MoCA: Montreal Cognitive Assessment
MRI: magnetic resonance imaging
PD: Parkinson disease
PD-D: Parkinson disease dementia
PD-MCI: Parkinson disease mild cognitive impairment
PD-NC: Parkinson disease normal cognition
PRISMA: Preferred Reporting Items for Systematic Review and Meta-Analyses
PROSPERO: International Prospective Register of Systematic Reviews
p-tau: phospho-microtubule-associated protein tau
QUADAS-2: Quality Assessment of Diagnostic Accuracy Studies-2
SNP: single-nucleotide polymorphism
SROC: summary receiver operator characteristic
SVM: support vector machine
TN: true negative
TP: true positive
UPDRS: Unified Parkinson’s Disease Rating Scale
XGBoost: extreme gradient boosting

## Data Availability

Data sharing is not applicable to this article as no datasets were generated or analyzed during this study.
