# Supplementary material for: The Role of Machine Learning in Cognitive Impairment in Parkinson Disease: Systematic Review and Meta-Analysis
Source: J Med Internet Res. 2025 Mar 14;27:e59649. doi: 10.2196/59649 (PMC11992493; doi:10.2196/59649)
Supplement: Multimedia Appendix 1 [file jmir_v27i1e59649_app1.docx]

# Supplementary Figures and Tables


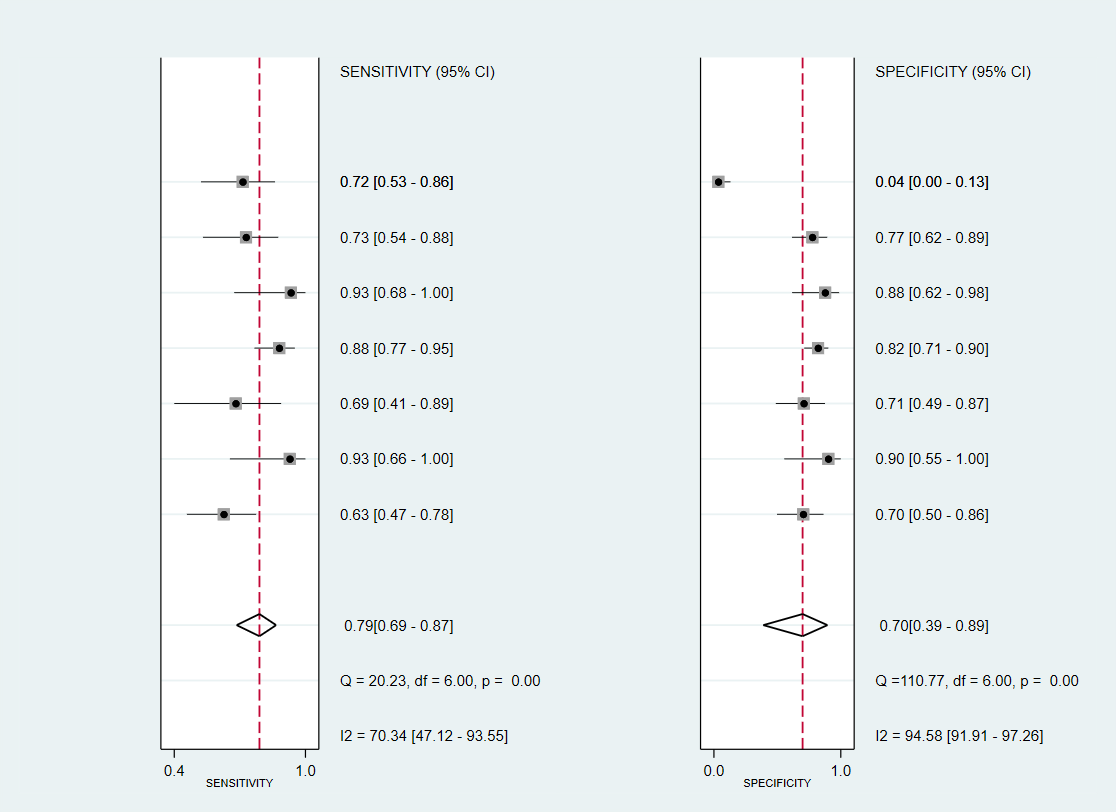


**Figure S1.** Forest plot of sensitivity and specificity for a single set (train or validation set).


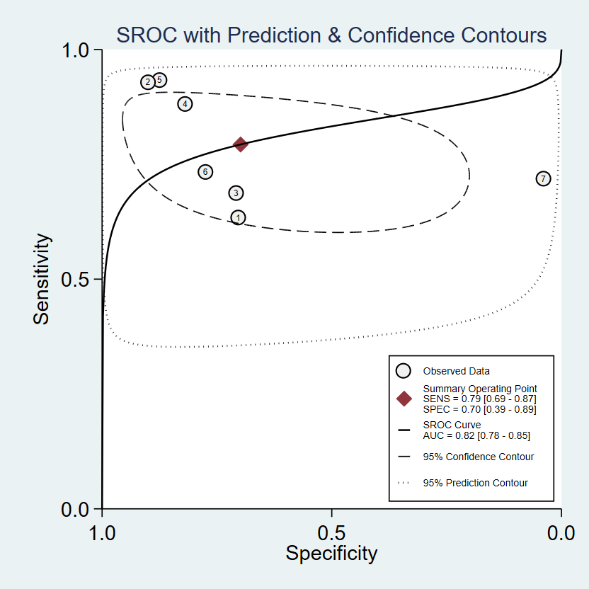


**Figure S2.** Summary receiver operating characteristic (SROC) curve for sensitivity and specificity in a single set (train or validation set).


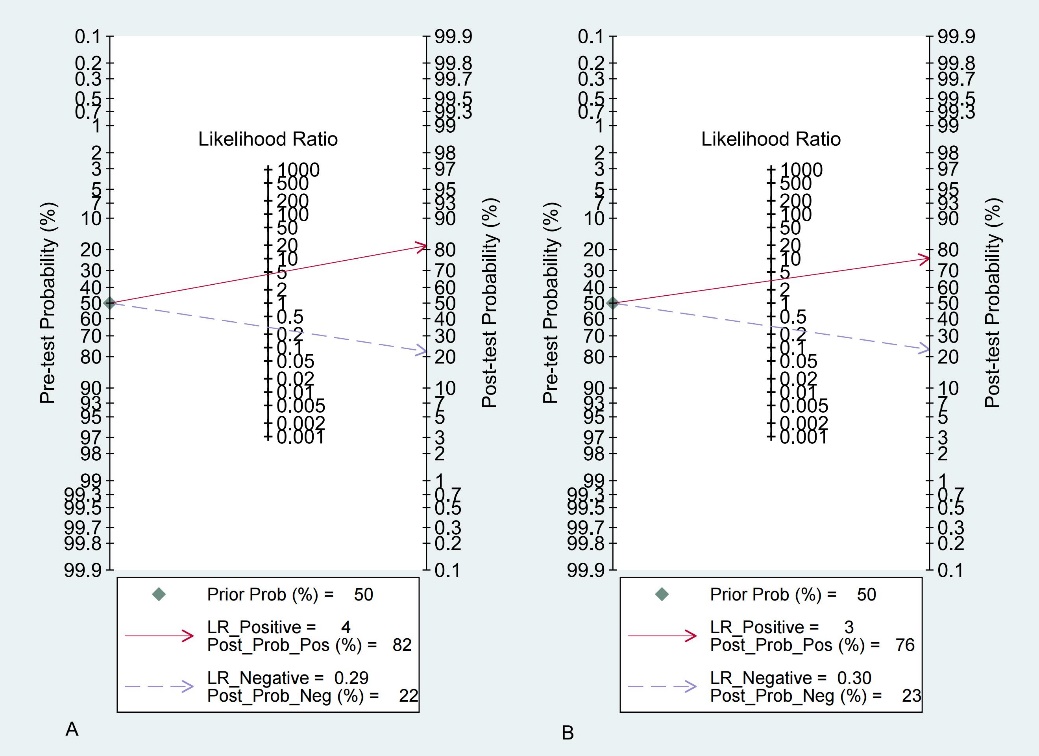


**Figure S3.** Fagan plot in train (A) and validation (B) set.

**
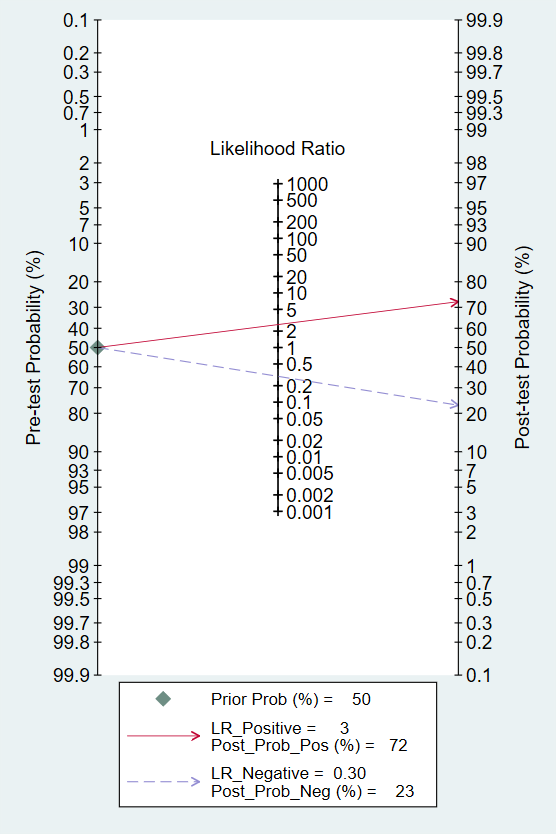
**

**Figure S4.** Fagan plot for a single set (train or validation set).

**
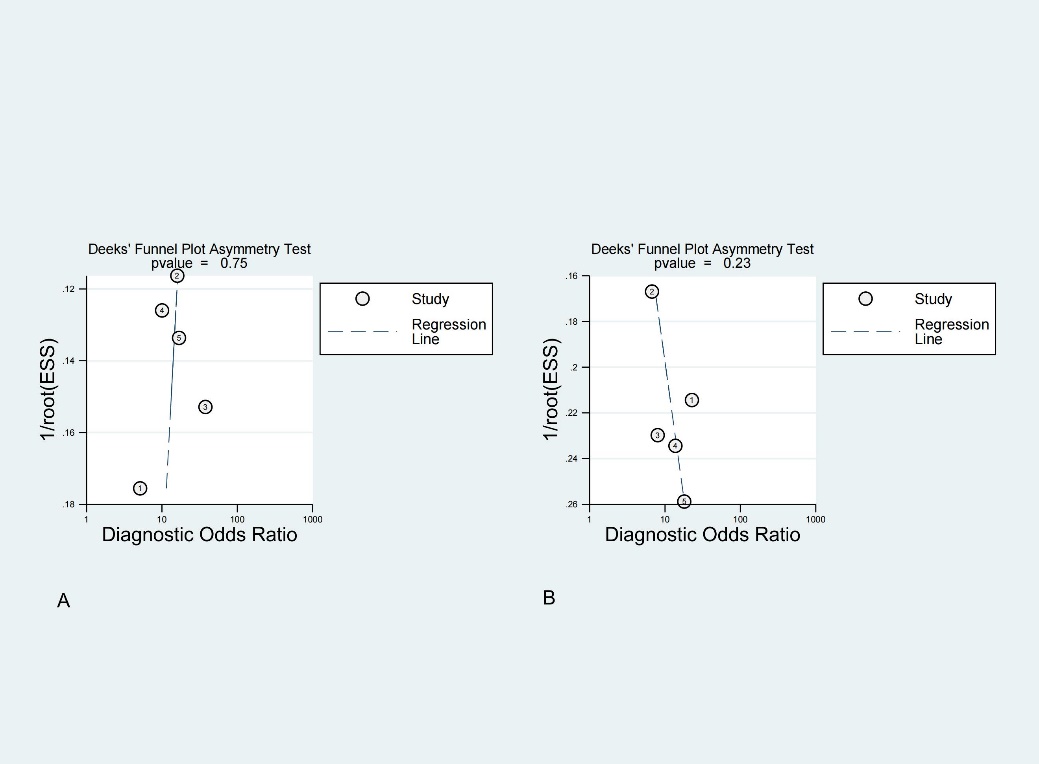
**

**Figure S5.** Deeks' funnel plot asymmetry test in train (A) and validation (B) set.

**
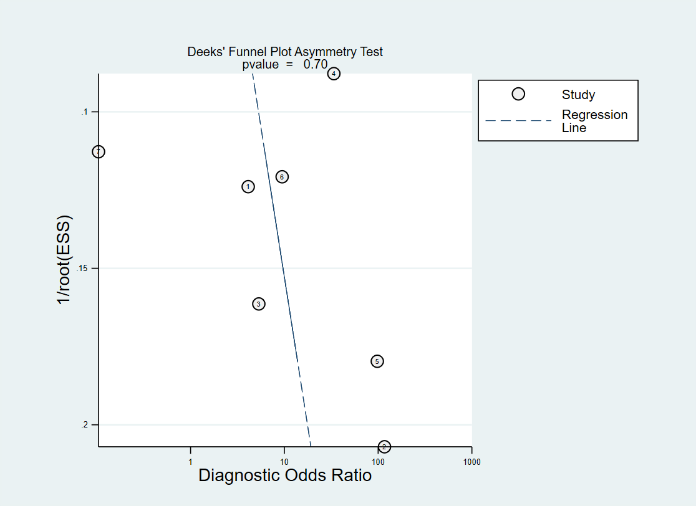
**

**Figure S6.** Deek’s test for a single set (train or validation set).


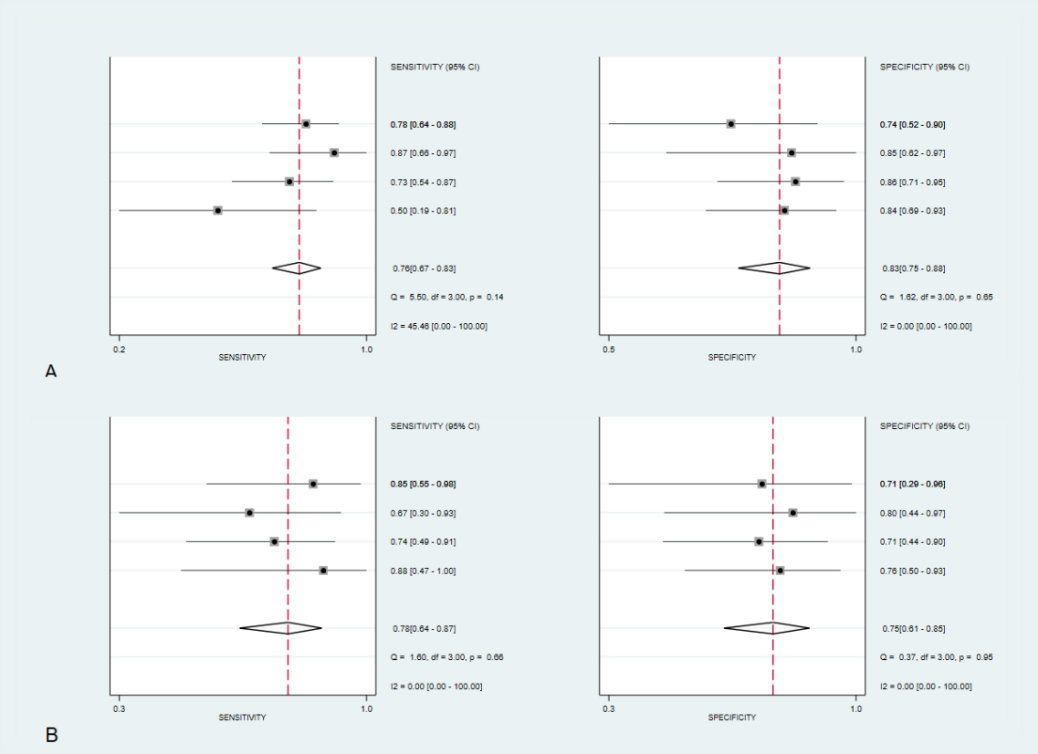


**Figure S7.** Forest plot of sensitivity and specificity in train (A) and calidation (B) set: sensitivity analysis of studies with high risk of bias exclusion.


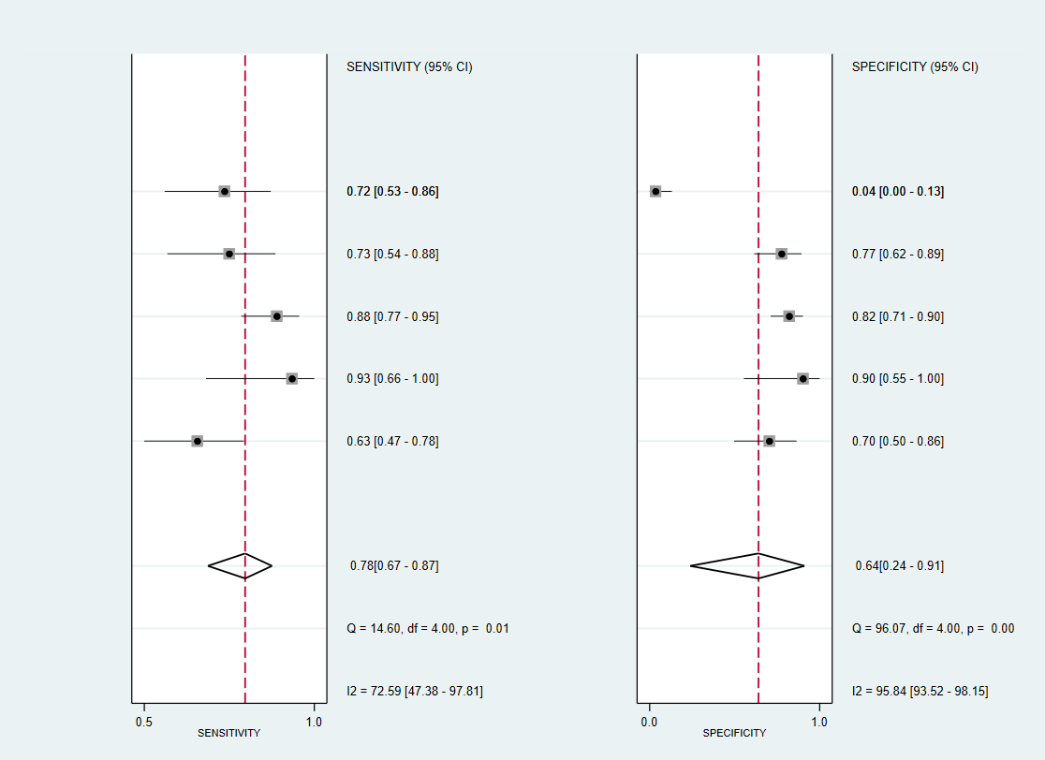


**Figure S8.** Forest plot of sensitivity and specificity for a single set (train or validation set): sensitivity analysis of studies with high risk of bias exclusion.


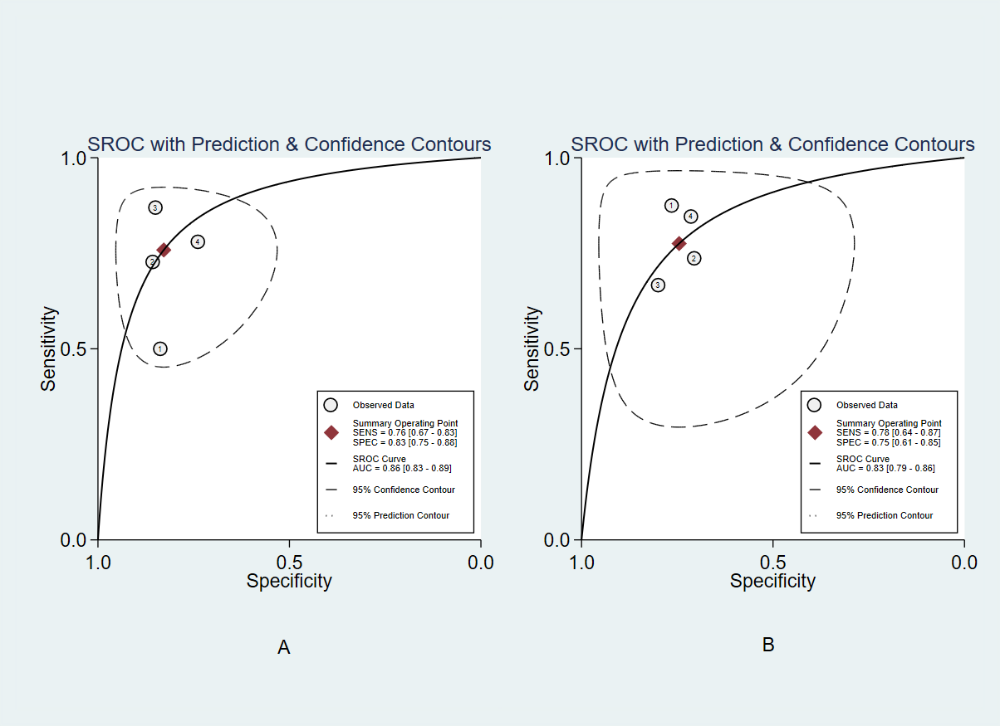


**Figure S9.** Summary receiver-operating characteristic curve of the sensitivity and specificity in train (A) and validation (B) set: sensitivity analysis after excluding studies with a high risk of bias.


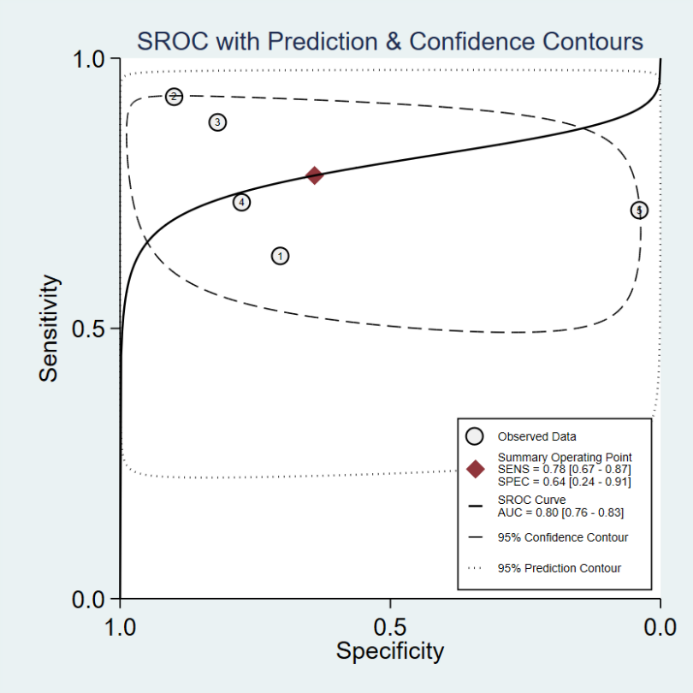


**Figure S10.** Summary receiver-operating characteristic curve for a single set (train or validation set): sensitivity analysis after excluding studies with a high risk of bias.


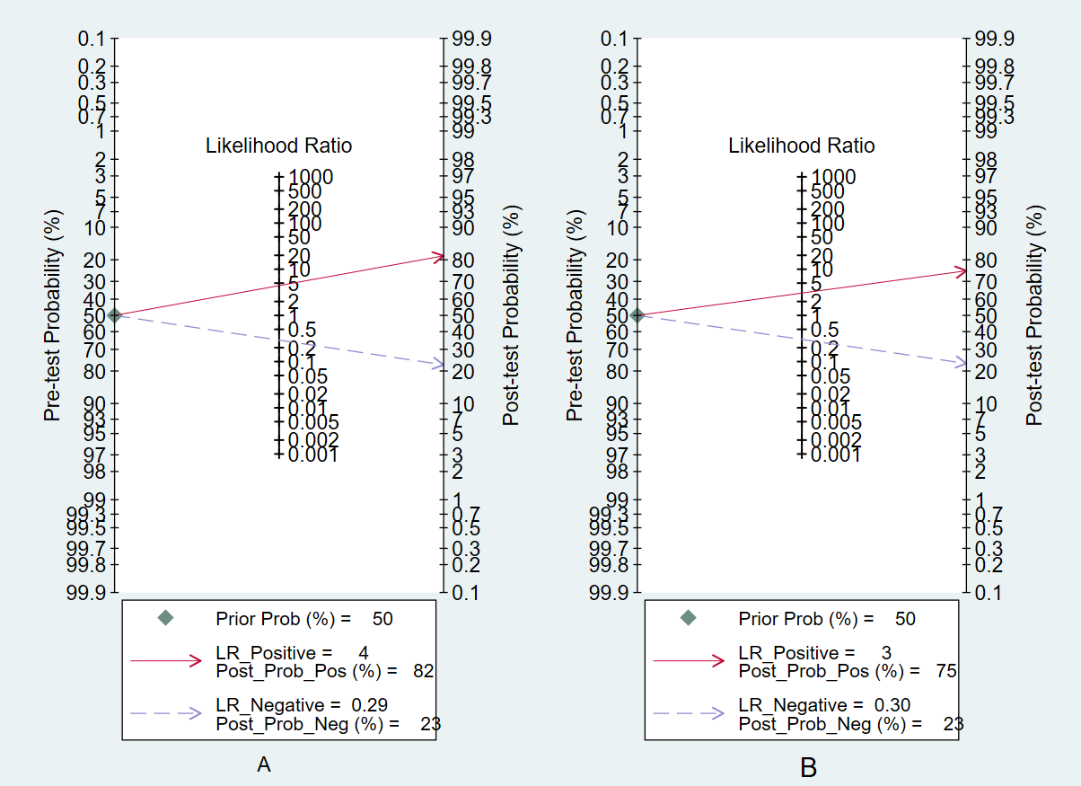


**Figure S11.** Fagan plot in train (A) and validation (B) set: sensitivity snalysis after excluding studies with a high risk of bias.


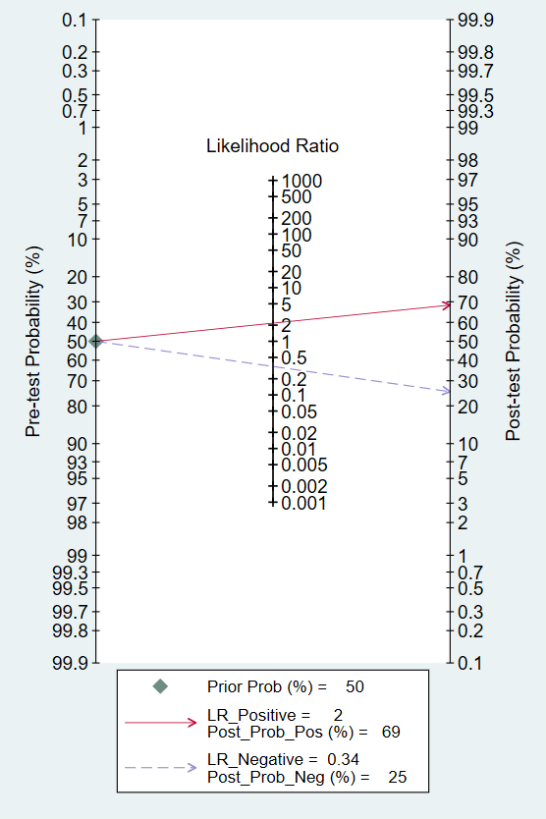


**Figure S12.** Fagan plot for a single set (train or validation set): sensitivity analysis after excluding studies with a high risk of bias.

**
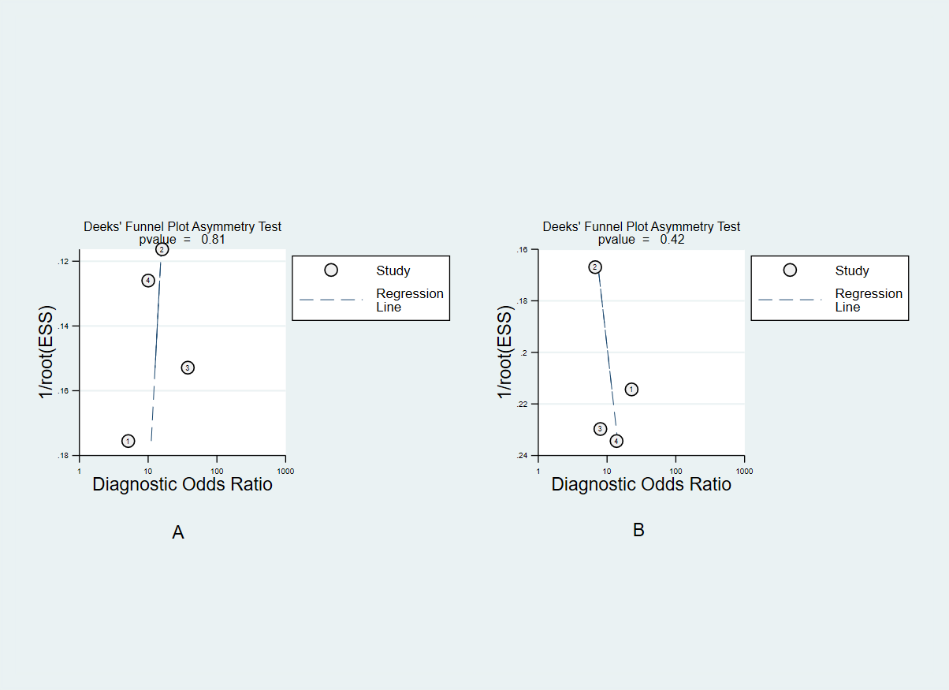
**

**Figure S13.** Deek’s test: sensitivity analysis: (A) train set and (B) validation Set.

**
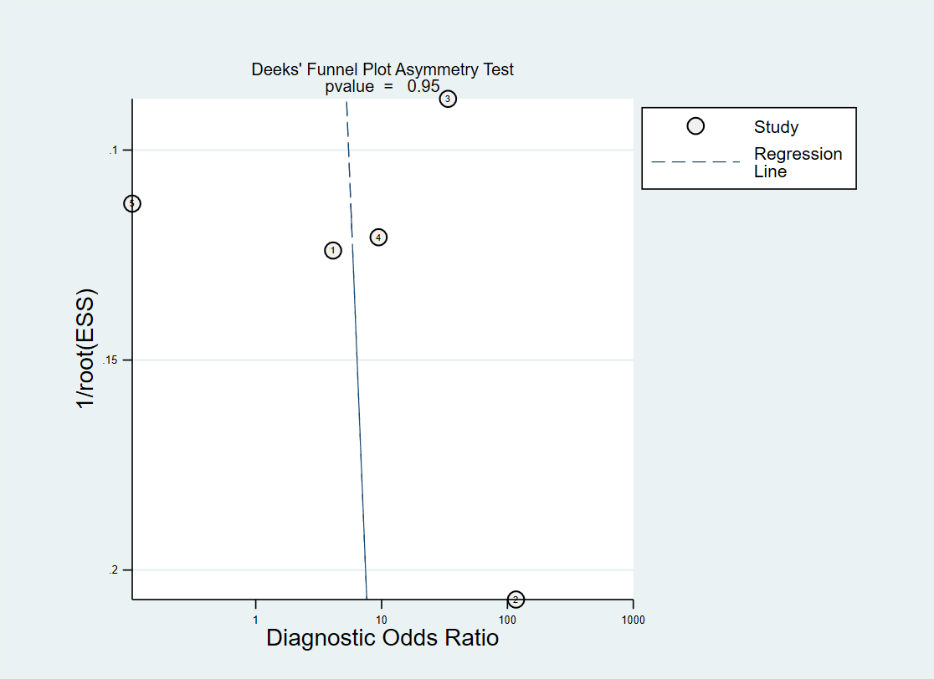
**

**Figure S14.** Deek’s test for a single set (train or validation set): sensitivity analysis.

## Table S1. Literature search strategy

1. Pubmed

| Search Number | Query |
| --- | --- |
| #1 | "Parkinson Disease"[MeSH Terms] |
| #2 | "Idiopathic Parkinson’s Disease"[Title/Abstract] OR "Lewy Body Parkinson s Disease"[Title/Abstract] OR "Parkinson s Disease Idiopathic"[Title/Abstract] OR "Parkinson s Disease Lewy Body"[Title/Abstract] OR "Parkinson Disease Idiopathic"[Title/Abstract] OR "Parkinson s Disease"[Title/Abstract] OR "Primary Parkinsonism"[Title/Abstract] OR "Paralysis Agitans"[Title/Abstract] |
| #3 | "Machine Learning"[MeSH Terms] |
| #4 | "Learning Machine"[Title/Abstract] OR "Transfer Learning"[Title/Abstract] OR "Learning Transfer"[Title/Abstract] |
| #5 | "Cognitive Dysfunction"[MeSH Terms] |
| #6 | "Cognitive dysfunctions"[Title/Abstract] OR "dysfunction Cognitive"[Title/Abstract] OR "Dysfunctions Cognitive"[Title/Abstract] OR "Cognitive Impairments"[Title/Abstract] OR "Cognitive Impairment"[Title/Abstract] OR "Impairment Cognitive"[Title/Abstract] OR "Impairments Cognitive"[Title/Abstract] OR "Cognitive Disorder"[Title/Abstract] OR "Cognitive Disorders"[Title/Abstract] OR "Disorder Cognitive"[Title/Abstract] OR "Disorders Cognitive"[Title/Abstract] OR "Mild Cognitive Impairment"[Title/Abstract] OR "Cognitive Impairment Mild"[Title/Abstract] OR "Cognitive Impairments Mild"[Title/Abstract] OR "Impairment mild Cognitive"[Title/Abstract] OR "Impairments mild Cognitive"[Title/Abstract] OR "Mild Cognitive Impairments"[Title/Abstract] OR "Cognitive decline"[Title/Abstract] OR "Cognitive Declines"[Title/Abstract] OR "Decline Cognitive"[Title/Abstract] OR "Declines Cognitive"[Title/Abstract] OR "Mental Deterioration"[Title/Abstract] OR "Deterioration Mental"[Title/Abstract] OR (("Deteriorate"[Title/Abstract] OR "Deteriorated"[Title/Abstract] OR "Deteriorates"[Title/Abstract] OR "Deteriorating"[Title/Abstract] OR "Deterioration"[Title/Abstract] OR "Deteriorations"[Title/Abstract] OR "Deteriorative"[Title/Abstract]) AND "Mental"[Title/Abstract]) OR "Mental Deteriorations"[Title/Abstract] |
| #7 | #1 OR #2 |
| #8 | #3 OR #4 |
| #9 | #5 OR #6 |
| #10 | #7 AND #8 AND #9 |

1. Web of science

| Search Number | Query |
| --- | --- |
| #1 | TS= ((Parkinson's Disease) OR (Parkinson Disease) OR (Idiopathic Parkinson's Disease) OR (Parkinson's Disease, Idiopathic) OR (Primary Parkinsonism) OR (Parkinsonism, Primary) OR (Paralysis Agitans)) OR (Lewy Body Parkinson Disease)) OR (Lewy Body Parkinson's Disease) OR (Parkinson's Disease, Lewy Body)) |
| #2 | TS= ((Machine Learning)) OR TS= (Learning, Machine)) OR TS= (Learning, Transfer)) OR TS= (Learning, Machine)) |
| #3 | (((((((((((((((((((((((((TS=(Cognitive Dysfunction)) OR TS=(Cognitive Dysfunctions)) OR TS=(Dysfunction, Cognitive)) OR TS=(Dysfunctions, Cognitive)) OR TS=(Cognitive Impairments)) OR TS=(Cognitive Impairment)) OR TS=(Impairment, Cognitive)) OR TS=(Impairments, Cognitive)) OR TS=(Cognitive Disorder)) OR TS=(Cognitive Disorders)) OR TS=(Disorder, Cognitive)) OR TS=(Disorders, Cognitive)) OR TS=(Mild Cognitive Impairment)) OR TS=(Cognitive Impairment, Mild)) OR TS=(Cognitive Impairments, Mild)) OR TS=(Impairment, Mild Cognitive)) OR TS=(Impairments, Mild Cognitive)) OR TS=(Mild Cognitive Impairments)) OR TS=(Cognitive Decline)) OR TS=(Cognitive Declines)) OR TS=(Decline, Cognitive)) OR TS=(Declines, Cognitive)) OR TS=(Mental Deterioration)) OR TS=(Deterioration, Mental)) OR TS=(Deteriorations, Mental)) OR TS=(Mental Deteriorations) and Preprint Citation Index (Exclude – Database) |
| #4 | #1 AND #2 AND 3 |

1. CoChrane

| Search Number | Query |
| --- | --- |
| #1 | MeSH descriptor: [Parkinson Disease] explode all trees |
| #2 | (Lewy Body Parkinson Disease):ti,ab,kw OR (Paralysis Agitans):ti,ab,kw OR (Parkinson Disease, Idiopathic):ti,ab,kw OR (Parkinson's Disease, Idiopathic):ti,ab,kw OR (Idiopathic Parkinson's Disease):ti,ab,kw (Word variations have been searched) |
| #3 | (Parkinson's Disease):ti,ab,kw OR (Primary Parkinsonism):ti,ab,kw OR (Idiopathic Parkinson Disease):ti,ab,kw OR (Parkinsonism, Primary):ti,ab,kw OR (Lewy Body Parkinson's Disease):ti,ab,kw (Word variations have been searched) |
| #4 | #1 OR #2 OR #3 |
| #5 | MeSH descriptor: [Machine Learning] explode all trees |
| #6 | (Learning, Machine):ti,ab,kw OR (Learning, Transfer):ti,ab,kw OR (Transfer Learning):ti,ab,kw |
| #7 | #5 OR #6 |
| #8 | MeSH descriptor: [Cognitive Dysfunction] explode all trees |
| #9 | (Mental Deterioration):ti,ab,kw OR (Cognitive Decline):ti,ab,kw OR (Declines, Cognitive):ti,ab,kw OR (Decline, Cognitive):ti,ab,kw OR (Mental Deteriorations):ti,ab,kw OR (Deterioration, Mental):ti,ab,kw OR (Deteriorations, Mental):ti,ab,kw OR (Cognitive Declines):ti,ab,kw OR (Disorder, Cognitive):ti,ab,kw OR (Dysfunction, Cognitive):ti,ab,kw OR (Cognitive Disorder):ti,ab,kw |
| #10 | #8 OR #9 |
| #11 | #4 AND #7 AND #10 |

1. Embase

| Search Number | Query |
| --- | --- |
| #1 | 'Parkinson Disease'/ exp |
| #2 | 'Idiopathic Parkinsonism'/exp OR 'Idiopathic Parkinsonism' OR 'Lewy Bodies of Parkinson Disease'/exp OR 'Lewy Bodies of Parkinson Disease' OR 'Lewy Bodies of Parkinson`s Disease'/exp OR 'Lewy Bodies of Parkinson`s disease' OR 'Lewy Bodies of Parkinsons Disease'/exp OR 'Lewy Bodies of Parkinsons Disease' OR 'Lewy Body Parkinson Disease'/exp OR 'Lewy body Parkinson Disease' OR 'Lewy body Parkinson`s Disease'/exp OR 'Lewy body Parkinson`s Disease' OR 'Lewy body Parkinsons Disease'/exp OR 'Lewy Body Parkinsons Disease' OR 'Paralysis Agitans'/exp OR 'Paralysis Agitans' OR 'Parkinson Dementia Complex'/exp OR 'Parkinson Dementia Complex' OR 'Parkinson`s Disease'/exp OR 'Parkinson`s Disease' OR 'Parkinsons Disease'/exp OR 'Parkinsons Disease' OR 'Primary Parkinsonism'/exp OR 'Primary Parkinsonism' OR 'Parkinson Disease'/exp OR 'Parkinson Disease' |
| #3 | #1 OR #2 |
| #4 | 'Machine Learning'/exp |
| #5 | 'Learning Machine'/exp OR 'Learning Machine' OR 'Learning Machines'/exp OR 'Learning Machines' OR 'Machine Learning'/exp OR 'Machine Learning' |
| #6 | #4 OR #5 |
| #7 | 'Cognitive defect'/exp |
| #8 | 'Cognition Disorder'/exp OR 'Cognition Disorder' OR 'Cognition Disorders'/exp OR 'Cognition Disorders' OR 'Cognitive Defects'/exp OR 'Cognitive Defects' OR 'Cognitive Deficit'/exp OR 'Cognitive Deficit' OR 'Cognitive Disability'/exp OR 'Cognitive Disability' OR 'Cognitive Disorder'/exp OR 'Cognitive Disorder' OR 'Cognitive Disorders'/exp OR 'Cognitive Disorders' OR 'Cognitive Dysfunction'/exp OR 'Cognitive Dysfunction' OR 'Cognitive Impairment'/exp OR 'Cognitive Impairment' OR 'Delirium, Dementia, Amnestic, Cognitive Disorders'/exp OR 'Delirium, Dementia, Amnestic, Cognitive Disorders' OR 'Overinclusion'/exp OR 'Overinclusion' OR 'Response Interference'/exp OR 'Response Interference' OR 'Cognitive Defect'/exp OR 'Cognitive Defect' |
| #9 | #7 OR #8 |
| #10 | #3 AND #6 AND #9 |

**Table S2. Characteristics of the included literature**

| Authors | Patient origin | Validation | HC | PD | Machine Learning | Predictors | Final model included predictors |
| --- | --- | --- | --- | --- | --- | --- | --- |
| Abos, A [1]  2017 | Single center | External | 38 | 95 | SVM, LR | Neuroimaging features | Brain connection-wise patterns of  connectivity |
| Almgren, H [2]  2023 | PPMI | Internal | 0 | 213 | SVR | Demographic, clinical, and biofluid markers | Age, gender, MoCA, GDS scores, STAI total scores, p-tau, t-tau, Aβ |
| Amboni, M [3]  2022 | Single center | Internal | 0 | 75 | J48, RF, ADA-B, KNN, SVM | Demographic, clinical, and neuroimaging features | model 1: age, psychiatric symptoms, depression, anxiety, apathy, sleep problems, FOG;  model 2A: PET imaging data; model 2B: PET imaging data+ model 1 |
| Andersson, S [4]  2021 | Single center | Internal | 145 | 347 | MLCLMM | Demographic, clinical, genetic, neuroimaging, and biofluid markers | Age, gender, education; UPDRS, MoCA, RBD, GDS, ApoE4, p-tau, t-tau, Aβ, α-syn, DAT density |
| Betrouni, N [5]  2019 | Multi-center | Internal | 0 | 118 | SVM, KNN | Demographic, and QEEG features | Age, gender, education, and peak frequency |
| Booth, S [6]  2022 | Multi-center | External | 18 | 76 | SVM | Neuroimaging features | Glucose metabolism scanned by FDG-PET |
| Byeon, H [7]  2017 | Multi-center | Internal | 35 | 26 | RF, LR, NB | Clinical features | SLVT, RCFT, K-CWST, TMT, and COWAT |
| Byeon, H [8]  2020 | Multi-center | Internal | 0 | 110 | LR, CART, RF | Clinical features | RBD, sleep disorders, K-MMSE, K-MoCA, GDS, CDR, K-IADL |
| Byeon, H [9]  2020 | Multi-center | Internal | 0 | 342 | RF, NB | Clinical features | K-MMSE, K-MoCA, CDR, UPDRS, K-IADL |
| Byeon, H [10]  2020 | Multi-center | Internal | 0 | 96 | RF, LR, DT | Clinical features | CDR, UPDRS, K-MMSE, K-MoCA |
| Byeon, H [11]  2021 | Multi-center | Internal | 320 | 48 | Boosting, RF, Bagging trees, | Clinical features | CSOA |
| Byeon, H [12]  2021 | Multi-center | Internal | 0 | 328 | Eps-SVR, Nu-SVR with four kernel functions (RBF, linear, polynomial, sigmoid) | Demographic, and clinical features | Age, age at diagnosis of PD, education level, family history of PD, pack year, coffee drinker, K-IADL, UPDRS, tremor, postural instability, atrial fibrillation, RBD, and depression |
| Cengiz, S [13]  2022 | Single center | Internal | 16 | 60 | KNN, SVM, Bagging trees | Neuroimaging features | Metabolic parameters and neuropsychological test scores were tested by 1H-MRSI |
| Chaturvedi, M[14]  2019 | Single center | Internal | 0 | 70 | RF | QEEG features | Frequency, PLI, frequency, and PLI |
| Chen, BY [15]  2023 | Single center | Internal | 0 | 120 | DT, RF, XGBoost | Neuroimaging features | Intra- and/or intervoxel metrics extracted from DTI |
| Chen, FZ [16]  2021 | Multi-center | Internal | 0 | 120 | Cox Regression | Clinical, neuroimaging, biofluid markers, and genetic variants | UPSIT score, DAT density, p-tau and α-syn, DDRGK1 rs55785911 |
| Chen, PH [17]  2022 | Single center | Internal | 0 | 42 | SVM-PCA | Clinical features, and biofluid markers | model 1: H-Y stage, IADL, Barthel index, UPDRS  model 2: model 1 + verbal fluency, digits forwards and backwards, TMT-B, α-syn, Aβ42, t-tau |
| Garcia, AM [18]  2021 | Single center | Internal | 40 | 40 | SVM | Clinical features | Prosodic, articulatory, and phonemic identifiability features. |
| Geraedts, VJ[19]  2021 | Single center | Internal | 0 | 112 | RF | QEEG features | EEG spectral characteristics, peak frequency |
| Harvey, J [20]  2022 | PPMI | Internal | 0 | 401 | Forest, SVM, Elastic Net, RF | Demographics, clinical, genetic, and biofluid markers | Gender, age of onset, education, duration of disease, family history of PD, MDS-UPDRS, rigidity score, H-Y stage, Aβ, p-tau, t-tau, α-syn, rs1241121663, rs35618164, rs391010565, GBA mutation status |
| Hogue, O [21]  2018 | PPMI | Internal | 0 | 351 | LR | Demographic, and clinical features | Education, verbal memory retention, right-sided bradykinesia, subjective report of cognitive impairment, and RBD |
| Huang, XF [22]  2024 | PPMI | Internal | 0 | 90 | LDA, SVM, KNN, NB | Demographic, clinical, and neuroimaging features | Age, UPDRS, the mean FL in different brain regions |
| Jeon, J [23]  2022 | PPMI | Internal | 0 | 397 | LR, SVM, RF | Demographic, and clinical features | Age, gender, years of education, handedness, duration of PD diagnosis, MoCA, SGD |
| Kawabata, K [24]  2018 | PPMI | Internal | 24 | 72 | ICA | Clinical, and neuroimaging features | resting‑state network, Mean regional FC values, ACE-R |
| Kibtia, H [25]  2020 | PPMI | Internal | 0 | 165 | SVM, RF | Clinical, neuroimaging, and biofluid markers | Age at onset, p-tau, α-syn, t- tau, mean putamen |
| Lin, H [26]  2021 | Single center, PPMI | Internal | 48 | 131 | RF | Neuroimaging features | SFG-STN SC, STG-IPL SC, hippocampus-thalamus SC, SFG-insula FC, IFG-PrG FC, PrG-insula FC, STG-precuneus FC, PhG-PoG FC, PoG-insula FC |
| Liu, GQ [27]  2017 | Multi-center | External | 0 | 2,482 | Cox regression | Demographic, clinical, and genetic features | GBA mutation status, age at onset, gender, years of education, MMSE, UPDRS, H-Y stage, and depression |
| Morales, A[28] 2013 | Single center, PPMI | Internal | 0 | 45 | NB, FSNB, CFS, SVM | Neuroimaging features | volumes of subcortical structure, cortical parcellation |
| Nguyen, AA [29] 2020 | PPMI | Internal | 42 | 116 | autoencoder neural network | Neuroimaging features | deformation-based morphometry values |
| Ortelli, P [30]  2022 | single center | Internal | 0 | 400 | QDA | Clinical features | CoMDA |
| Ostertag, C [31] 2023 | ANDI and PPMI | External | 200 | 400 | Deep neural network | Clinical, and neuroimaging features | Multimodal3DSiameseNet |
| Ramezani, M [32] 2021 | Single center | Internal | 0 | 101 | SVR | Clinical, neuroimaging, features, and genetic features | Gender, education, UPDRS, Cortical thickness, right parahippocampal cortex, and right caudate volume, rs994280 |
| Salmanpour, M [33] 2019 | PPMI | External | 158 | 492 | LOLIMOT, RBF, MLp-BP, LASSOLAR, RF, RNN, BRR, DT, PAR, thiel-sen Regression | Demographic, and clinical features | 93 features including age, gender, family history, UPDRS score, UPSIT |
| Shin, NY [34]  2021 | Single center | External | 0 | 141 | RF, SVM*LASSO | Clinical, and neuroimaging features | Cortical thickness |
| Tang, C [35]  2021 | PPMI | Internal | 0 | 108 | LASSO, COX | Clinical features | MoCA, SDMT, LNS, HVLT immediate recall, HVLT delayed recall, HVLT recognition hits, UPDRS total, RBDSQ, and rad-score |
| Yu ZY [36]  2023 | Single center | Internal | 30 | 70 | SVM | Neuroimaging features | 6 major association fiber tracts, one major commissural fiber tract; and one major projection fiber tract |
| Zhang, J [37]  2020 | Single center | Internal | 20 | 93 | SVM | Neuroimaging, and QEEG features | Based on SBM abstracting QEGG and imaging features |
| Zhang, J [38]  2021 | Single center | Internal | 0 | 71 | SVM | Neuroimaging, and QEEG features | Cortical thickness, volume and mean curvature characteristics |

Note: PD, Parkinson’s disease; HC, healthy controls; SVM, support vector machine; LR, logistic regression; SVR, support vector regression; RF, random forest; KNN, k-nearest neighbor; ADA-B, Ada boost; MLCLMM, multivariate latent class linear mixed model; NB, naïve Bayes; CART, Classification and Regression Tree; DT, decision tree; LASSO, least absolute shrinkage and selection operator; XGBoost, eXtreme Gradient Boosting; PCA, principal component analysis; LDA, Latent Dirichlet Allocation; ICA, Independent Component Analysis; Cox, cox proportional-hazards model; FSNB, feature selection naïve Bayes; CFS, correlation-based feature selection; LASSOLAR, least absolute shrinkage and selection operator least angle regression; RNN, recurrent neural networks; BRR, binary neural networks; QDA, quadratic discriminant analysis; MoCA, Montreal Cognitive Assessment; GDS, Geriatric Depression Scale; MMSE, mini-mental state examination; STAI, State-trait anxiety inventory; p-tau, phosphorylated tau; Aβ, amyloid beta; FOG, freezing of gait; PET, Positron Emission Computed Tomography; UPDRS, Unified Parkinson’s Disease Rating Scale; RBD, REM (rapid eyes movement) behavior disorder; DTA, dopamine transporter; QEEG, quantitative electroencephalography; FDG-PET, brain fluorodeoxyglucose-PET; SLVT, Seoul verbal learning test; RCFT, Rey complex figure test; K-CWST, Korean color word Stroop test; COWAT, controlled oral word association test; K-MMSE, Korean-mini-mental state examination; K-MoCA, Korean-Montreal Cognitive Assessment; CDR, Clinical Dementia Rating; K-IADL, Korean-instrumental activity of daily living; CSOA, cognition scale for olde adults; BF, radial basis function; PLI, phase lag index; DTI ,diffusion tensor image; UPSIT, University of Pennsylvania Smell Identification Test; CSF, cerebrospinal fluid; TMT, trail making test; FL, fiber length; SGD , The Strengths and Difficulties Questionnaire; FC, fiber cross-section; ACE-R, Addenbrooke’ s cognitive examination-revised; H-Y stage, Hoehn & Yahr stage; RVLT, Rey's Auditory Verbal Learning Test; SFG, superior frontal gyrus; STG, superior temporal gyrus; IPL, inferior parietal lobule; IFG, inferior frontal gyrus; PrG, precentral gyrus; PhG, parahippocampal gyrus; PoG, postcentral gyrus; HVLT, Hopkins Verbal Learning Test; SDMT, Symbol Digit Modalities Test; LNS, Letter-Number Sequencing; ADNI, Alzheimer's Disease Neuroimaging Initiative; PPMI, Parkinson's Disease Progression Markers Initiative; ROI, region of interest; CoMDA, cognition in movement disorders assessment.

**Table S3. Risk of bias status of each included article.**

| Authors | Patient selection | Index test | Reference standard | Flow and timing | Data management | Total |
| --- | --- | --- | --- | --- | --- | --- |
| Abos, A [1]  2017 | low | low | low | low | low | low |
| Almgren, H [2]  2023 | low | unclear | unclear | low | low | unclear |
| Amboni, M [3]  2022 | low | low | low | low | low | low |
| Andersson, S [4]  2021 | low | unclear | unclear | low | low | unclear |
| Betrouni, N [5]  2019 | low | unclear | high | low | low | high |
| Booth, S [6]  2022 | low | low | low | low | low | low |
| Byeon, H [7]  2017 | low | low | high | low | low | high |
| Byeon, H [8]  2020 | low | low | low | low | low | low |
| Byeon, H [9]  2020 | unclear | low | low | low | low | unclear |
| Byeon, H [10]  2020 | low | low | unclear | low | low | unclear |
| Byeon, H [11]  2021 | high | low | high | low | low | high |
| Byeon, H [12]  2021 | unclear | low | high | low | low | high |
| Cengiz, S [13]  2022 | low | low | low | low | low | low |
| Chaturvedi, M[14]  2019 | low | low | low | low | low | low |
| Chen, BY [15]  2023 | low | low | low | low | low | low |
| Chen, FZ [16]  2021 | low | low | unclear | low | low | unclear |
| Chen, PH [17]  2022 | low | low | high | low | low | high |
| Garcia, AM [18]  2021 | high | low | high | low | low | high |
| Geraedts, VJ[19]  2021 | low | low | high | low | low | high |
| Harvey, J [20]  2022 | low | low | unclear | low | low | unclear |
| Hogue, O [21]  2018 | low | low | high | low | low | high |
| Huang, XF [22]  2024 | low | low | low | low | low | low |
| Jeon, J [23]  2022 | low | low | low | low | low | low |
| Kawabata, K [24]  2018 | low | low | high | high | low | high |
| Kibtia, H [25]  2020 | low | low | high | low | low | high |
| Lin, H [26]  2021 | low | low | low | low | low | low |
| Liu, GQ [27]  2017 | unclear | low | low | low | low | unclear |
| Morales, A[28]  2013 | low | low | high | low | low | high |
| Nguyen, AA [29]  2020 | low | low | high | low | low | high |
| Ortelli, P [30]  2022 | unclear | low | high | low | low | high |
| Ostertag, C [31]  2023 | low | low | high | low | low | high |
| Ramezani, M [32]  2021 | high | low | high | low | low | high |
| Salmanpour, M [33]  2019 | unclear | high | high | low | low | high |
| Shin, NY [34]  2021 | low | low | unclear | low | low | unclear |
| Tang, C [35]  2021 | low | low | high | low | low | high |
| Yu ZY [36]  2023 | unclear | low | low | low | low | unclear |
| Zhang, J [37]  2020 | low | low | low | low | low | low |
| Zhang, J [38]  2021 | high | low | low | low | low | high |

**References**

1. Abos A, Baggio HC, Segura B, Garcia-Diaz AI, Compta Y, Marti MJ, Valldeoriola F, Junque C. Discriminating cognitive status in Parkinson’s disease through functional connectomics and machine learning. Sci Rep 2017 Mar 28;7. doi: 10.1038/srep45347

2. Almgren H, Camacho M, Hanganu A, Kibreab M, Camicioli R, Ismail Z, Forkert ND, Monchi O. Machine learning-based prediction of longitudinal cognitive decline in early Parkinson’s disease using multimodal features. Sci Rep 2023 Aug 14;13(1). doi: 10.1038/s41598-023-37644-6

3. Amboni M, Ricciardi C, Adamo S, Nicolai E, Volzone A, Erro R, Cuoco S, Cesarelli G, Basso L, D’Addio G, Salvatore M, Pace L, Barone P. Machine learning can predict mild cognitive impairment in Parkinson’s disease. Front Neurol 2022 Nov 17;13. doi: 10.3389/fneur.2022.1010147

4. Andersson S, Josefsson M, Stiernman LJ, Rieckmann A. Cognitive Decline in Parkinson’s Disease: A Subgroup of Extreme Decliners Revealed by a Data-Driven Analysis of Longitudinal Progression. Front Psychol 2021;12:729755. PMID:34566817

5. Betrouni N, Delval A, Chaton L, Defebvre L, Duits A, Moonen A, Leentjens AFG, Dujardin K. Electroencephalography-based machine learning for cognitive profiling in Parkinson’s disease: Preliminary results. Mov Disord 2019 Feb;34(2):210–217. doi: 10.1002/mds.27528

6. Booth S, Park KW, Lee CS, Ko JH. Predicting cognitive decline in Parkinson’s disease using FDG-PET-based supervised learning. J Clin Invest 2022 Oct 17;132(20):e157074. PMID:36040832

7. Byeon H, Jin H, Cho S. Development of Parkinson’s disease dementia prediction model based on verbal memory, visuospatial memory, and executive function. J Med Imaging Health Inform 2017;7(7):1517–1521. doi: 10.1166/jmihi.2017.2196

8. Byeon H. Application of Machine Learning Technique to Distinguish Parkinson’s Disease Dementia and Alzheimer’s Dementia: Predictive Power of Parkinson’s Disease-Related Non-Motor Symptoms and Neuropsychological Profile. J Pers Med 2020 Jun;10(2). doi: 10.3390/jpm10020031

9. Byeon H. Best early-onset Parkinson dementia predictor using ensemble learning among Parkinson’s symptoms, rapid eye movement sleep disorder, and neuropsychological profile. World J Psychiatry 2020 Nov 19;10(11):245–259. doi: 10.5498/wjp.v10.i11.245

10. Byeon H. Is the random forest algorithm suitable for predicting parkinson’s disease with mild cognitive impairment out of parkinson’s disease with normal cognition? Int J Environ Res Public Health 2020;17(7). doi: 10.3390/ijerph17072594

11. Byeon H. Exploring Parkinson’s Disease Predictors based on Basic Intelligence Quotient and Executive Intelligence Quotient. Int J Adv Comput Sci Appl 2021 Apr;12(4):106–111.

12. Byeon H. Predicting the severity of parkinson’s disease dementia by assessing the neuropsychiatric symptoms with an svm regression model. Int J Environ Res Public Health 2021;18(5):1–9. doi: 10.3390/ijerph18052551

13. Cengiz S, Arslan DB, Kicik A, Erdogdu E, Yildirim M, Hatay GH, Tufekcioglu Z, Ulug AM, Bilgic B, Hanagasi H, Demiralp T, Gurvit H, Ozturk-Isik E. Identification of metabolic correlates of mild cognitive impairment in Parkinson’s disease using magnetic resonance spectroscopic imaging and machine learning. Magn Reson Mater Phys Biol Med 2022 Dec;35(6):997–1008. doi: 10.1007/s10334-022-01030-6

14. Chaturvedi M, Bogaarts JG, Kozak Cozac VV, Hatz F, Gschwandtner U, Meyer A, Fuhr P, Roth V. Phase lag index and spectral power as QEEG features for identification of patients with mild cognitive impairment in Parkinson’s disease. Clin Neurophysiol Off J Int Fed Clin Neurophysiol 2019 Oct;130(10):1937–1944. PMID:31445388

15. Chen B, Xu M, Yu H, He J, Li Y, Song D, Fan GG. Detection of mild cognitive impairment in Parkinson’s disease using gradient boosting decision tree models based on multilevel DTI indices. J Transl Med 2023 May 8;21(1). doi: 10.1186/s12967-023-04158-8

16. Chen F, Li Y, Ye G, Zhou L, Bian X, Liu J. Development and Validation of a Prognostic Model for Cognitive Impairment in Parkinson’s Disease With REM Sleep Behavior Disorder. Front Aging Neurosci 2021;13:703158. PMID:34322014

17. Chen P-H, Hou T-Y, Cheng F-Y, Shaw J-S. Prediction of Cognitive Degeneration in Parkinson’s Disease Patients Using a Machine Learning Method. Brain Sci 2022 Aug;12(8). doi: 10.3390/brainsci12081048

18. García AM, Arias-Vergara T, C. Vasquez-Correa J, Nöth E, Schuster M, Welch AE, Bocanegra Y, Baena A, Orozco-Arroyave JR. Cognitive Determinants of Dysarthria in Parkinson’s Disease: An Automated Machine Learning Approach. Mov Disord 2021;36(12):2862–2873. doi: 10.1002/mds.28751

19. Geraedts VJ, Koch M, Contarino MF, Middelkoop HAM, Wang H, Van Hilten JJ, Bäck THW, Tannemaat MR. Machine learning for automated EEG-based biomarkers of cognitive impairment during Deep Brain Stimulation screening in patients with Parkinson’s Disease. Clin Neurophysiol 2021 May;132(5):1041–1048. doi: 10.1016/j.clinph.2021.01.021

20. Harvey J, Reijnders RA, Cavill R, Duits A, Kohler S, Eijssen L, Rutten BPF, Shireby G, Torkamani A, Creese B, Leentjens AFG, Lunnon K, Pishva E. Machine learning-based prediction of cognitive outcomes in de novo Parkinson’s disease. Npj Park Dis 2022 Nov 7;8(1). doi: 10.1038/s41531-022-00409-5

21. Hogue O, Fernandez HH, Floden DP. Predicting early cognitive decline in newly-diagnosed Parkinson’s patients: A practical model. Parkinsonism Relat Disord 2018 Nov;56:70–75. PMID:29936131

22. Huang X, He Q, Ruan X, Li Y, Kuang Z, Wang M, Guo R, Bu S, Wang Z, Yu S, Chen A, Wei X. Structural connectivity from DTI to predict mild cognitive impairment in de novo Parkinson’s disease. Neuroimage-Clin 2024;41. doi: 10.1016/j.nicl.2023.103548

23. Jeon J, Kim K, Baek K, Chung SJ, Yoon J, Kim YJ. Accuracy of Machine Learning Using the Montreal Cognitive Assessment for the Diagnosis of Cognitive Impairment in Parkinson’s Disease. J Mov Disord 2022 May;15(2):132–139. PMID:35670022

24. Kawabata K, Watanabe H, Hara K, Bagarinao E, Yoneyama N, Ogura A, Imai K, Masuda M, Yokoi T, Ohdake R, Tanaka Y, Tsuboi T, Nakamura T, Hirayama M, Ito M, Atsuta N, Maesawa S, Naganawa S, Katsuno M, Sobue G. Distinct manifestation of cognitive deficits associate with different resting-state network disruptions in non-demented patients with Parkinson’s disease. J Neurol 2018 Mar;265(3):688–700. doi: 10.1007/s00415-018-8755-5

25. Kibtia H, Abdullah S, Bustamam A. Comparison of Random Forest and Support Vector Machine for Prediction of Cognitive Impairment in Parkinson’s Disease. 2020. doi: 10.1063/5.0030332

26. Lin H, Liu Z, Yan W, Zhang D, Liu J, Xu B, Li W, Zhang Q, Cai X. Brain connectivity markers in advanced Parkinson’s disease for predicting mild cognitive impairment. Eur Radiol 2021 Dec;31(12):9324–9334. PMID:34109485

27. Liu G, Locascio JJ, Corvol J-C, Boot B, Liao Z, Page K, Franco D, Burke K, Jansen IE, Trisini-Lipsanopoulos A, Winder-Rhodes S, Tanner CM, Lang AE, Eberly S, Elbaz A, Brice A, Mangone G, Ravina B, Shoulson I, Cormier-Dequaire F, Heutink P, van Hilten JJ, Barker RA, Williams-Gray CH, Marinus J, Scherzer CR, HBS, CamPaIGN, PICNICS, PROPARK, PSG, DIGPD, PDBP. Prediction of cognition in Parkinson’s disease with a clinical-genetic score: a longitudinal analysis of nine cohorts. Lancet Neurol 2017 Aug;16(8):620–629. PMID:28629879

28. Morales DA, Vives-Gilabert Y, Gomez-Anson B, Bengoetxea E, Larranaga P, Bielza C, Pagonabarraga J, Kulisevsky J, Corcuera-Solano I, Delfino M. Predicting dementia development in Parkinson’s disease using Bayesian network classifiers. Psychiatry Res-Neuroimaging 2013 Aug 30;213(2):92–98. doi: 10.1016/j.pscychresns.2012.06.001

29. Nguyen AA, Maia PD, Gao X, F Damasceno P, Raj A. Dynamical Role of Pivotal Brain Regions in Parkinson Symptomatology Uncovered with Deep Learning. Brain Sci 2020 Jan 30;10(2):73. PMID:32019067

30. Ortelli P, Ferrazzoli D, Versace V, Cian V, Zarucchi M, Gusmeroli A, Canesi M, Frazzitta G, Volpe D, Ricciardi L, Nardone R, Ruffini I, Saltuari L, Sebastianelli L, Baranzini D, Maestri R. Optimization of cognitive assessment in Parkinsonisms by applying artificial intelligence to a comprehensive screening test. Npj Park Dis 2022 Apr 11;8(1). doi: 10.1038/s41531-022-00304-z

31. Ostertag C, Visani M, Urruty T, Beurton-Aimar M. Long-term cognitive decline prediction based on multi-modal data using Multimodal3DSiameseNet: transfer learning from Alzheimer’s disease to Parkinson’s disease. Int J Comput Assist Radiol Surg 2023 May;18(5):809–818. doi: 10.1007/s11548-023-02866-6

32. Ramezani M, Mouches P, Yoon E, Rajashekar D, Ruskey JA, Leveille E, Martens K, Kibreab M, Hammer T, Kathol I, Maarouf N, Sarna J, Martino D, Pfeffer G, Gan-Or Z, Forkert ND, Monchi O. Investigating the relationship between the SNCA gene and cognitive abilities in idiopathic Parkinson’s disease using machine learning. Sci Rep 2021 Mar 1;11(1):4917. doi: 10.1038/s41598-021-84316-4

33. Salmanpour MR, Shamsaei M, Saberi A, Setayeshi S, Klyuzhin IS, Sossi V, Rahmim A. Optimized machine learning methods for prediction of cognitive outcome in Parkinson’s disease. Comput Biol Med 2019 Aug;111. doi: 10.1016/j.compbiomed.2019.103347

34. Shin N-Y, Bang M, Yoo S-W, Kim J-S, Yun E, Yoon U, Han K, Ahn KJ, Lee S-K. Cortical Thickness from MRI to Predict Conversion from Mild Cognitive Impairment to Dementia in Parkinson Disease: A Machine Learning-based Model. Radiology 2021 Aug;300(2):390–399. doi: 10.1148/radiol.2021203383

35. Tang C, Zhao X, Wu W, Zhong W, Wu X. An individualized prediction of time to cognitive impairment in Parkinson’s disease: A combined multi-predictor study. Neurosci Lett 2021 Sep 25;762:136149. PMID:34352339

36. Yu Z, Pang H, Yu H, Wu Z, Ding Z, Fan G. Segmental disturbance of white matter microstructure in predicting mild cognitive impairment in idiopathic Parkinson’s disease: An individualized study based on automated fiber quantification tractography. Park Relat Disord 2023 Oct;115. doi: 10.1016/j.parkreldis.2023.105802

37. Zhang J, Li Y, Gao Y, Hu J, Huang B, Rong S, Chen J, Zhang Y, Wang L, Feng S, Wang L, Nie K. An SBM-based machine learning model for identifying mild cognitive impairment in patients with Parkinson’s disease. J Neurol Sci 2020 Nov 15;418. doi: 10.1016/j.jns.2020.117077

38. Zhang J, Gao Y, He X, Feng S, Hu J, Zhang Q, Zhao J, Huang Z, Wang L, Ma G, Zhang Y, Nie K, Wang L. Identifying Parkinson’s disease with mild cognitive impairment by using combined MR imaging and electroencephalogram. Eur Radiol 2021 Oct;31(10):7386–7394. doi: 10.1007/s00330-020-07575-1
